# Mouse and rat BDNF gene structure and expression revisited

**DOI:** 10.1002/jnr.21139

**Published:** 2006-12-05

**Authors:** Tamara Aid, Anna Kazantseva, Marko Piirsoo, Kaia Palm, Tõnis Timmusk

**Affiliations:** Department of Gene Technology, Tallinn University of TechnologyTallinn, Estonia

**Keywords:** BDNF, exon, promoter, DNA methylation, histone deacetylation, calcium, kainic acid

## Abstract

Brain-derived neurotrophic factor (BDNF) has important functions in the development of the nervous system and in brain plasticity-related processes such as memory, learning, and drug addiction. Despite the fact that the function and regulation of rodent BDNF gene expression have received close attention during the last decade, knowledge of the structural organization of mouse and rat BDNF gene has remained incomplete. We have identified and characterized several mouse and rat BDNF transcripts containing novel 5′ untranslated exons and introduced a new numbering system for mouse and rat BDNF exons. According to our results both mouse and rat BDNF gene consist of eight 5′ untranslated exons and one protein coding 3′ exon. Transcription of the gene results in BDNF transcripts containing one of the eight 5′ exons spliced to the protein coding exon and in a transcript containing only 5′ extended protein coding exon. We also report the distinct tissue-specific expression profiles of each of the mouse and rat 5′ exon-specific transcripts in different brain regions and nonneural tissues. In addition, we show that kainic acid-induced seizures that lead to changes in cellular Ca^2+^ levels as well as inhibition of DNA methylation and histone deacetylation contribute to the differential regulation of the expression of BDNF transcripts. Finally, we confirm that mouse and rat BDNF gene loci do not encode antisense mRNA transcripts, suggesting that mechanisms of regulation for rodent and human BDNF genes differ substantially. © 2006 Wiley-Liss, Inc.

Brain-derived neurotrophic factor (BDNF) is a member of the neurotrophin family consisting of small secreted proteins that play important roles in the development of the nervous system in vertebrates (for recent reviews see Bibel and Barde, 2000; Binder and Scharfman, 2004; Chao et al., 2006). BDNF supports the survival and differentiation of specific populations of embryonic neurons in vivo, and growing evidence indicates that BDNF is also involved in several functions in adulthood, including neuronal homeostasis and brain plasticity-related processes such as memory, learning (Tyler et al., 2002; Yamada et al., 2002), and drug addiction (Bolanos and Nestler, 2004). Alterations in BDNF expression in specific neuron subpopulations contribute to various pathologies, including depression, epilepsy, and Alzheimer's, Huntington's, and Parkinson's diseases (Bibel and Barde, 2000; Murer et al., 2001; Binder and Scharfman, 2004; Castren, 2004; Cattaneo et al., 2005; Russo-Neustadt and Chen, 2005).

In addition to BDNF, the neurotrophin family includes nerve growth factor, neurotrophin-3, and neurotrophin-4/5 (Binder, 2004). All neurotrophins bind to p75^NGFR^ receptor but selectively interact with their individual high-affinity protein kinase receptors of the *trk* (tropomyosin-related kinase) family (Kaplan and Miller, 2000; Chao, 2003; Teng and Hempstead, 2004). BDNF mediates its biological effects via TrkB and p75^NGFR^ receptors. Binding of mature BDNF protein to TrkB and p75^NGFR^ promotes cell survival, neurite outgrowth, synaptic transmission, plasticity, and cell migration (Dechant and Barde, 2002). Uncleaved precursor BDNF protein (pro-BDNF) has altered binding characteristics and distinct biological activity in comparison with mature BDNF protein (Lee et al., 2001; Teng et al., 2005).

In mouse and rat, BDNF mRNA is expressed throughout development and differentially in adult tissues (Ernfors et al., 1990; Hofer et al., 1990; Hohn et al., 1990). In the brain, BDNF mRNA and protein expression becomes detectable during embryonic development, reaching the highest levels by days 10–14 postnatally and decreasing thereafter. In the adult animal, BDNF is expressed throughout the brain, with the highest levels in the neurons of hippocampus (Ernfors et al., 1990; Hofer et al., 1990; Kawamoto et al., 1996; Conner et al., 1997; Yan et al., 1997). Neuronal BDNF expression is affected by many stimuli, such as γ-aminobutyric acid (GABA)-ergic and glutamatergic neurotransmission and membrane depolarization through calcium-mediated pathways (Zafra et al., 1990, 1991; Ghosh et al., 1994; Shieh and Ghosh, 1999; West et al., 2001). BDNF gene expression is controlled by multiple activity-dependent and tissue-specific promoters. Four BDNF promoters have been previously identified in rat (Metsis et al., 1993; Timmusk et al., 1993; Timmusk et al., 1995), each driving the transcription of BDNF mRNAs containing one of the four 5′ noncoding exons (I, II, III, or IV) spliced to the common 3′ coding exon. Several transcription factors contributing to the regulation of BDNF promoters have been characterized. Among these factors are cAMP-responsive element binding protein (CREB; Shieh et al., 1998; Tao et al., 1998; Tabuchi et al., 2002) and upstream stimulatory factors 1/2 (USF1/2; Tabuchi et al., 2002; Chen et al., 2003b), which regulate BDNF promoters I and III. In addition, calcium-responsive transcription factor (CaRF) has been found to mediate BDNF transcription through binding to BDNF promoter III upon neuronal activation (Tao et al., 2002). Chromatin remodeling by DNA methylation and histone deacetylation also plays an important role in cell-specific and activity-dependent regulation of BDNF gene by recruiting global repressors such as REST/NRSF to promoter II (Palm et al., 1998; Timmusk et al., 1999; Zuccato et al., 2003) and MeCP2 to promoter III (Chen et al., 2003a; Martinowich et al., 2003).

Unraveling the regulation of BDNF gene expression is important for understanding its contribution to nervous system function and pathology. Provided that BDNF actions are most frequently modeled in rodents, detailed knowledge of the structural organization of rodent BDNF genes would be imperative. We undertook this study to specify the structure and expression of BDNF gene in mouse and rat. We show that rodent BDNF gene structure and expression are more complex than initially characterized (Timmusk et al., 1993) and that novel, as yet unidentified regulatory sequences may contribute to cell-specific and activity-dependent regulation of rodent BDNF expression.

## MATERIALS AND METHODS

### DNA and Amino Acid Sequence Analysis

Mouse and rat BDNF gene structure in silico analysis was performed using genomic, mRNA and EST databases (http://www.ncbi.nlm.nih.gov and http://genome. ucsc.edu). Alignment tools available at http://www.ncbi. nlm.nih.gov as well as software provided by the BIIT group at the University of Tartu, Estonia, were used for homology searches and analysis. AntiHunter software (available at http://bio.ifom-firc.it/ANTIHUNTER/) was used to search for opposite-strand transcripts in mouse and rat BDNF genomic region.

### RNA Isolation, cDNA Synthesis, RT-PCR

Total RNA from developing and adult mouse and rat total brain and brain regions and nonneural tissues was purified by RNAwiz (Ambion, Austin, TX) as recommended by the manufacturer. DNase treatment of total RNA was perfomed by using a Turbo DNA-Free Kit (Ambion) according to the manufacturer's instructions. Five micrograms of total RNA from different tissues was used for first-strand synthesis using oligo(dT) and SuperScript III First-Strand synthesis system (Invitrogen, Carlsbad, CA). To analyze expression of BDNF transcripts, reverse primer specific for 3′ BDNF coding exon and forward primers specific for 5′ noncoding exons were used. To identify homologues of human antisense BDNF exons in mouse and rat, primers were designed corresponding to mouse and rat BDNF genomic regions that showed significant homology with human exons. Total RNA was normalized to the expression of ubiquitously expressed HPRT gene. All primers used in the study are listed below, where m designates mouse, r, rat; h, human; for, forward; rev, reverse; and Arabic numbers BDNF exons as follows: mrBDNFI, GTGTGACCTGAGCAGTGGGCAAAGGA; mrBDNFII, GGAAGTGGAAGAAACCGTCTAGAGCA; mBDNFIII, GCTTTCTATCATCCCTCCCCGAGAGT; rBDNFIII, CCTTTCTATTTTCCCTCCCCGAGAGT; mrBDNFIV, CTCTGCCTAGATCAAATGGAGCTTC; mrBDNFV, CTCTGTGTAGTTTCATTGTGTGTTC; mBDNFVI, GCTGGCTGTCGCACGGTTCCCATT; rBDNFVI, GCTGGCTGTCGCACGGTCCCCATT; mrBDNFVII, CCTGAAAGGGTCTGCGGAACTCCA; mrBDNFVIII, GTGTGTGTCTCTGCGCCTCAGTGGA; mBDNFIXA, CCCAAAGCTGCTAAAGCGGGAGGAAG; rBDNFIXA, CCAGAGCTGCTAAAGTGGGAGGAAG; hmrHPRT for, GATGATGAACCAGGTTATGAC; hmrHPRTrev, GTCCTTTTCACCAGCAAGCTTG; and mrBDNFrev, GAAGTGTACAAGTCCGCGTCCTTA.

To analyze expression of mouse and rat exons I–IV-, exon VI-, and exon IXA-specific transcripts, cDNA was amplified in a total volume of 25 μl with 35 cycles of PCR using HotFire polymerase system (Solis BioDyne, Estonia). An annealing temperature of 60°C was used for all primer combinations. Because of relatively low expression levels of BNDF mRNAs containing exons V, VII, and VIII, a more robust HotStartTaq Master Mix kit (Qiagen, Chatsworth, CA) was used for cDNA amplification for 40–45 PCR cycles. All RT-PCR reactions were performed in triplicate. PCR products were resolved in 1.2% agarose gel and visualized by staining with ethidium bromide. PCR fragments were subsequently excised from the gel, cloned by using pCRII-TOPO cloning system (Invitrogen), and subjected to sequence analysis.

### 5′ RACE Analyses of Transcription Initiation Sites

To determine the transcription start sites of novel BDNF transcripts, 5′ rapid amplification of cDNA ends (RACE) was performed by using the GeneRacer kit (Invitrogen) according to the manufacturer's instructions. PCR amplification was performed with a HotStartTaq Master Mix kit (Qiagen) and GeneRacer 5′ forward primer and reverse primers specific for exons III, V, VII, VIII, and IXA. Then, nested PCR was performed to increase the specificity and sensitivity of RACE by using GeneRacer 5′ nested primer and nested primers specific for exons III, V, VII, VIII, and IXA. RACE products were analyzed in a 2% gel and cloned into the pCRII-Topo vector (Invitrogen) for sequence analysis. Primers used for RACE analysis are listed below: rBDNFIIIRACE, TCAATGAAGCATCCAGCCCGGCA; rBDNFIIINested, CGGAACTCTCGGGGAGGGAA AATA; rBDNFVRACE, GAACACACAATGAAACTACACAGAG; rBDNFVIIRACE, CTAAAGAGGTGCGCTGGATGGACAGAG; rBDNFVIINested, GGACCTGGAGTTCCGCAGACCCTTT; rBDNF VIIIRACE, CCATTTTCAGCAATCGTTTGTTCAGC; rBDNFVIIINested, GAGACACACACCACAGCCTTTCTC; rBDNFIXARACE, GAGTAAACGGTTTCTAAGCAA GTG; and rBDNFIXANested, CTTCCTCCCACTTTAGCAGCTCTG.

### Cell Culture and Animal Experiments

Rat glioma C6 and mouse neuroblastoma Neuro2A cells were plated 16 hr before treatment in DMEM (Invitrogen) containing 10% fetal bovine serum (FBS), 100 U/ml penicillin, 100 μg/ml streptomycin). Trichostatin A (TSA) and 5-aza-2′-deoxycytidine (5AzadC) were purchased from Sigma-Aldrich (St. Louis, MO). Neuro-2A and C6 cells were treated for 48 hr with 5AzadC (1 μM) or with TSA (333 nM) to analyze the effects of 5AzadC and TSA on the expression of BDNF.

Adult male Sprague-Dawley rats were injected with the glutamate analog kainic acid as previously described (Metsis et al., 1993). Animals were sacrificed 1, 3, 6, 12, and 24 hr posttreatment. Total RNA from hippocampi was extracted by using RNAwiz RNA Isolation Reagent (Ambion) according to the manufacturer's recommendations. All animal experiments were performed according to the norms of the local Ethical Committee of Animal Experimentation.

## RESULTS

### New Nomenclature for Mouse and Rat BDNF Gene

**Fig. 1:**
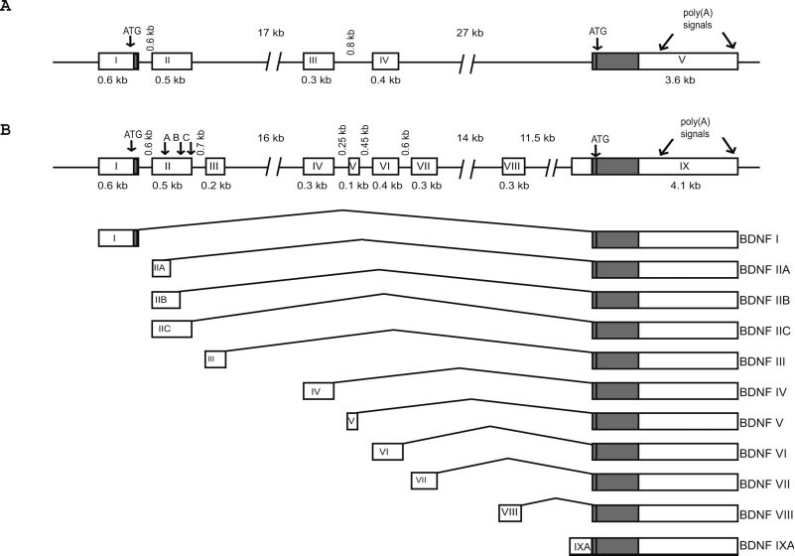
Exon/intron structure and alternative transcripts of mouse and rat BDNF genes. **A:** Rat BDNF gene structure as described by Timmusk et al. (1993). Exons are shown as boxes and introns are shown as lines. **B:** The new arrangement of exons and introns of mouse and rat BDNF genes as determined by analyzing genomic and mRNA sequence data using bioinformatics, 5′ RACE, and RT-PCR. The schematic representation of BDNF transcripts in relation to the gene is shown below the gene structure. Protein coding regions are shown as solid boxes and untranslated regions are shown as open boxes. Each of the eight 5′ untranslated exons is spliced to the common 3′ protein coding exon IX. In addition, transcription can be initiated in the intron before the protein coding exon, which results in IXA transcripts containing 5′ extended coding exon. Each transcription unit may use one of the two alternative polyadenylation signals in the 3′ exon (arrows). For exon II, three different transcript variants, IIA, IIB, and IIC, are generated as a result of using alternative splice-donor sites in exon II (arrows marked A, B, and C).

BDNF gene is transcribed from multiple promoters located upstream of distinct 5′ noncoding exons to produce a heterogeneous population of BDNF mRNAs. Although this conserved feature of BDNF has been described for several species, including human (Liu et al., 2005), mouse (Hayes et al., 1997), rat (Timmusk et al., 1993), and zebrafish (Heinrich and Pagtakhan, 2004), detailed analyses of rodent BDNF gene structure have not been performed. In rat, four 5′ noncoding exons (I–IV) that are spliced to the common 3′ coding exon (Fig. 1A) have previously been identified (Timmusk et al., 1993). For mouse, only homologues of rat BDNF exons I and II have been reported (Hayes et al., 1997). In silico analysis of mouse and rat BDNF gene structure performed in the present study showed that BDNF exons III and IV are present and expressed in mouse as well. Moreover, a number of EST and mRNA sequences aligned to the locations of potential novel BDNF exons and the respective sequences turned out to be highly conserved in rat and mouse genome. Furthermore, analysis of BDNF 5′ RACE products from human hippocampal RNA revealed additional novel exons (Kazantseva et al., unpublished), the sequences of which were also conserved in mouse and rat genomes. Identification of rodent BDNF transcripts containing novel exons by RT-PCR and subsequent cloning and sequencing confirmed the bioinformatic analyses data. Together our results show that both rat and mouse BDNF gene contains eight 5′ noncoding exons and one 3′ protein coding exon. All exon–intron junctions display conventional splice-donor and -acceptor sites. A new nomenclature was assigned to mouse and rat BDNF exons (Fig. 1B). In both mouse and rat genomes, the locations of novel BDNF exons are as following: exon III (corresponding to rat exon Ia described by Bishop et al., 1994) is located 0.6 kb downstream of previously described exon II, exon V is 0.25 kb downstream of exon IV (exon IV is the former exon III according to Timmusk et al., 1993), exon VII is located 0.6 kb downstream of exon VI (exon VI corresponds to exon IV in Timmusk et al. 1993), exon VIII is 13.5 kb upstream of the protein coding exon, and exon IXA is a 5′ extended variant of the protein coding exon (Fig. 1B). Homology of human and rodent BDNF 5′ exons ranges from 95% to 45%, reaching 95% for exon I, 93% for exon II, 62% for exon III, 91% for exon IV, 86% for exon VI (corresponds to exon V in human according to Liu et al., 2005), and 45% for exon VII (corresponds to exon VIA in human according to Liu et al., 2005). All exons that have been defined in human (Liu et al., 2005) are also expressed in mouse and rat, except for human exons VIIB and VIII. Rodent exons V, VIII, and IXA have not been previously described in human (Liu et al., 2005), but according to our data these exons are expressed in human as well (Kazantseva et al., unpublished). Rat BDNF gene has been suggested to undergo cryptic splicing within exon II (Timmusk et al., 1995). In agreement with the recently updated version of GenBank's submission (AY057907), our results show that usage of alternative splice donor sites (A, B, and C in Fig. 1B) within BDNF exon II leads to three different exon II transcript variants in both in mouse and rat.

### Expression Analysis of Mouse and Rat BDNF Transcripts

**Fig. 2:**
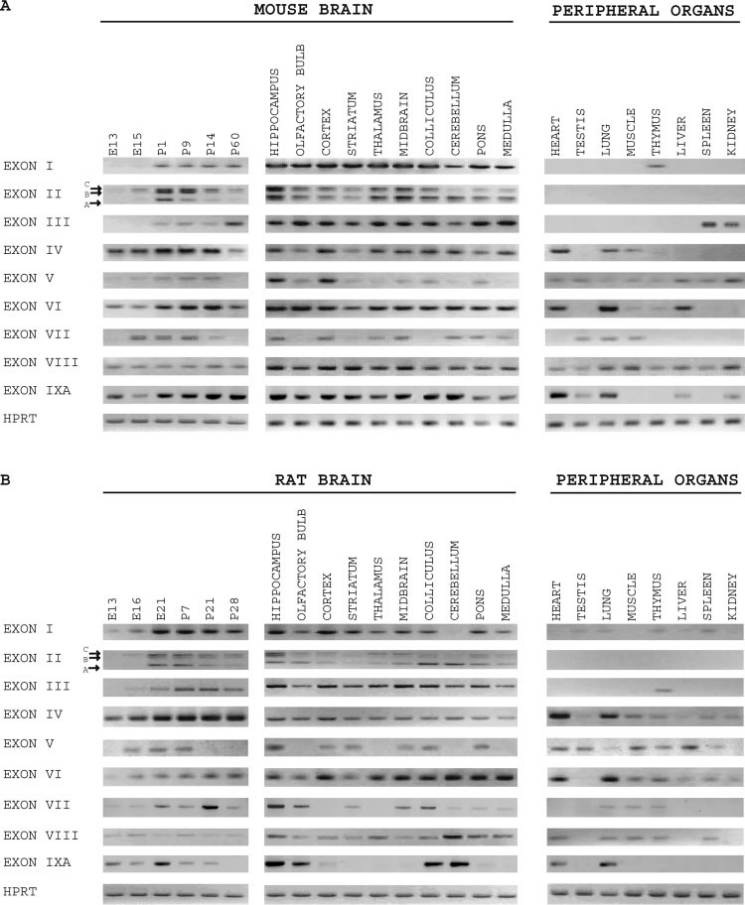
Expression analysis of mouse and rat BDNF mRNAs. Semiquantitive RT-PCR analysis of tissue-specific expression of mouse (**A**) and rat (**B**) BDNF transcripts and control HPRT mRNA was performed in developing and adult brain and in peripheral organs. E, embryonic day; P, postnatal day.

Rat BDNF transcripts containing exons I, II, IV (former III), and VI (former IV) and their tissue-specific expression profiles have previously been described (Timmusk et al., 1993), whereas there are no data on the expression of the novel rat BDNF exons V, VII, VIII, and IXA, and only limited data are available on the expression patterns of rat exon III (Bishop et al., 1994). Furthermore, although promoter regions upstream of mouse BDNF exons I and II have been described (Hayes et al., 1997), no data are available for the expression of mouse BDNF transcripts containing exons I–IXA. In the present work, RT-PCR analysis of the expression profiles of all BDNF transcripts was carried out in developing and adult brain as well as in peripheral tissues of mouse and rat (Fig. 2).

Expression of rat exon I BDNF mRNA, which was previously described as a brain-specific transcript (Timmusk et al., 1993), was also observed at low levels in several nonneural tissues, including testis, lung, thymus, liver, and spleen (Fig. 2B). Expression of mouse exon I transcripts was detected in addition to brain only in thymus (Fig. 2A). In adult mouse and rat brain, BDNF exon I mRNAs were expressed in all regions studied, with the lowest levels in cerebellum. In developing mouse and rat brain, low levels of BDNF exon I transcripts were expressed at embryonic (E) days 13 and 15, the expression levels peaked at postnatal (P) day 1 in mouse and embryonic day 21 in rat and decreased slightly during postnatal development (Fig. 2A,B). BDNF exon II mRNA splice variants A, B, and C revealed differential expression patterns in brain both in mouse and in rat, and their expression was not detected in peripheral tissues. In cerebellum, exon IIA transcript was the most abundant; in hippocampus, all three exon II splice variants were expressed at similar levels. Overall brain-specific expression pattern of mouse and rat novel BDNF exon III transcripts resembled that of BDNF exon II (Fig. 2A,B). In murine nonneural tissues, low levels of exon III transcripts were detected only in spleen and kidney, and, in rat, exon III transcripts were detected in thymus. BDNF exon IV and exon VI mRNAs (formerly exons III and IV, Timmusk et al., 1993) were observed at significant levels in developing mouse and rat brain already at E13, the earliest developmental stage studied. Both in mouse and in rat, BDNF exon IV and exon VI mRNA levels increased gradually during embryonic and postnatal development and decreased slightly in adult brain. In adult brain, exon IV and exon VI transcripts were detected in all analyzed brain regions both in mouse and in rat. Exon IV and exon VI transcripts exhibited wide patterns of expression in mouse and rat nonneural tissues, with the highest levels in heart and lung (Fig. 2A,B).

In both mouse and rat, BDNF novel exons V, VII, and VIII, were expressed at relatively low levels during brain development, broadly in adult peripheral tissues, and differentially in adult brain regions (Fig. 2A,B). In spite of the fact that mouse BDNF mRNA containing exons VII and VIII in the same transcript has been submitted to NCBI GenBank (AY231132), we failed to detect similar mRNAs in any of mouse or rat tissue studied.

Expression of the novel BDNF exon IXA transcripts was detected in rodent brain during embryonic development as well as in adulthood. In mouse adult brain, exon IXA-containing transcripts were expressed at similar levels in all brain regions (Fig. 2A), whereas, in rat adult brain, exon IXA expression was detected at high levels in hippocampus, olfactory bulb, colliculus, and cerebellum and at lower levels in cortex and pons (Fig. 2B). In rodent nonneural tissues, relatively high levels of exon IXA transcripts were observed in heart and lung.

### Identification of the Transcription Start Sites for BDNF New Exons III, V, VII, and VIII in Rat

**Fig. 3:**
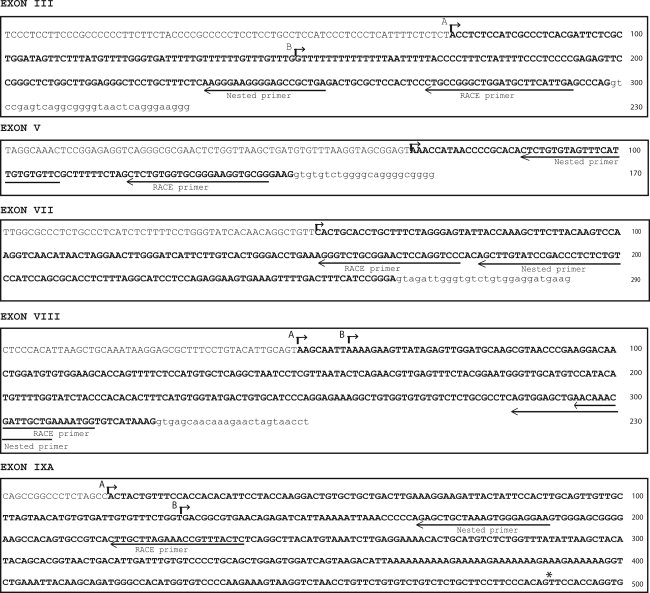
Identification of the transcription start sites for BDNF new exon III, V, VII, VIII, and IXA mRNAs in rat. 5′ Rapid amplification of cDNA ends (5′ RACE) was performed to determine the transcription start sites for novel BDNF transcripts. Major transcription initiation sites (short arrows) are located at 152 bp and 230 bp for exon III (arrows marked A and B), at 81 bp for exon V and at 277 bp and 286 bp for exon VIII (arrows marked A and B) upstream of the 3′ end of the respective exon and at 476 bp and 363 bp upstream of the major splice acceptor site of the coding exon (asterisk) for exon IXA. For exon VII, the 5′end of the longest EST is shown as a putative transcription initiation site, because 5′ RACE did not result in any specific products as a result of very low levels of exon VII transcripts. Exon sequences are in boldface; intron sequences are in lowercase letters. The locations of primers that were used in 5′ RACE are indicated with long arrows.

The transcription initiation sites for rat BDNF exons I, II, IV, and VI have been determined earlier (Timmusk et al., 1993). To identify the transcription start sites for novel BDNF transcripts, 5′ rapid amplification of cDNA ends (5′ RACE) from rat hippocampal RNA was performed by using antisense primers specific for exons III, V, VII, VIII, and IXA. Sequencing analysis of different RACE clones showed that major transcription initiation sites are located at 152 bp and 230 bp for exon III, at 81 bp for exon V, at 277 bp and 286 bp for exon VIII upstream of the 3′ end of the respective exon, and at 476 bp and 363 bp for exon IXA upstream of the major splice site of this exon (Fig. 3). None of the identified 5′ exons contains upstream open reading frames, so the usage of these 5′UTRs will apparently not affect amino acid composition of the protein product. Because of the very low expression levels, we failed to map the transcription start site for rodent BDNF exon VII. However, 5′ RACE analysis of rodent exon VII homologue in human showed that transcription initiation site for this exon is located at 285 bp upstream of its 3′ end (Kazantseva et al., unpublished). These data strongly suggest that, similarly to BDNF exon I, II, IV, and VI mRNAs (Timmusk et al., 1993), novel exon III, V, VII, VIII, and IXA mRNAs are also transcribed from separate promoters.

### Differential Regulation of BDNF Expression by DNA Methylation and Histone Deacetylation

Provided that methylation of the cytosine residues in the CpG dinucleotides in genome and posttranslational modifications of histones in the nucleosome establishes epigenetic codes for gene regulation in different tissues, including nervous system (Hsieh and Gage, 2004; Ballas and Mandel, 2005), we investigated the potential role of chromatin structure on transcriptional activity of BDNF promoters. By treating rat C6 glioma cells and mouse Neuro2A neuroblastoma cells with the DNA methyltransferase inhibitor 5AzadC or with the histone deacetylase (HDAC) inhibitor TSA for 48 hr, we examined the role of DNA methylation and histone acetylation, respectively, in the regulation of BDNF gene expression.

**Fig. 4:**
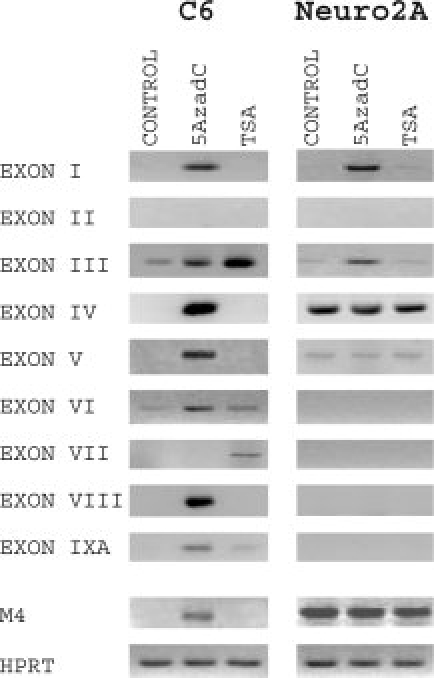
Differential regulation of BDNF gene expression by DNA methylation and histone deacetylation. The role of DNA methylation in transcriptional activity of BDNF promoters was investigated by treating rat C6 glioma and mouse Neuro2A neuroblastoma cells with 1 μM 5-Aza-deoxycytidine (5AzadC) for 48 hr. The effects of inhibition of histone deacetylation was studied by treating Neuro2A and C6 cells with 300 nM trichostatin A (TSA) for 48 hr. Muscarinic acetylcholine receptor M4 gene and constitutive hypoxanthine-phosphoribosyltransferase (HPRT) gene were used as reference genes.

We observed robust activation of the expression of BDNF exon I and IV as well as novel exon V, VIII, and IXA transcripts in rat C6 glioma cells after 5AzadC treatment (Fig. 4). Expression of exons III and VI in C6 cells was moderately induced by inhibition of DNA methylation. Expression of BDNF exon I and exon III transcripts in Neuro2A cells was significantly induced following 5AzadC treatment, whereas there was no change in the levels of other BDNF mRNAs (Fig. 4). In Neuro2A cells, TSA treatment failed to relieve repression of any of the BDNF promoters. However, in C6 cells, inhibition of histone deacetylation by TSA increased the levels of BDNF exon III, exon VII, and exon IXA transcripts. Muscarinic acetylcholine receptor gene M4 was used as a reference because its expression has been shown to be regulated by 5AzadC in various cell lines, other than C6 and Neuro2A, in a cell-type-specific manner (Lunyak et al., 2002; Wood et al., 2003). Our findings suggest that DNA methylation and histone deacetylation could play a role in silencing of BDNF gene in a promoter- and cell-specific manner both in C6 and Neuro2A cells.

### Activity-Dependent Regulation of Rat BDNF Exon-Specific mRNAs in the Hippocampus by Kainic Acid-Induced Seizures

**Fig. 5:**
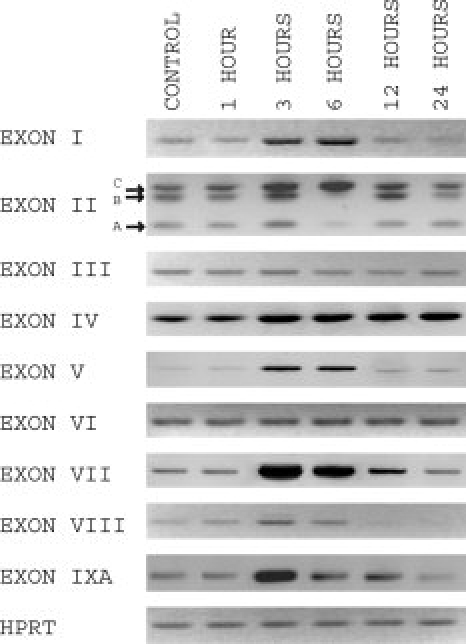
Activity-dependent regulation of BDNF exon-specific mRNAs in rat hippocampus by kainic acid-induced seizures. The effect of kainate-induced seizures on the expression of different BDNF transcripts in the hippocampus of adult rat brain was examined. Adult rats were injected subcutaneously with kainic acid (8 mg per kg body weight) and sacrificed 1, 3, 6, 12, and 24 hr posttreatment. Total RNA was extracted, and semiquantitative RT-PCR was performed. Untreated rat hippocampus RNA was used as a control.

Glutamate analogue kainic acid induces a rise in intracellular Ca^2+^ levels and differential activation of four previously characterized BDNF promoters in the hippocampus and cerebral cortex of adult rat brain (Timmusk et al., 1993). We examined whether expression of the BDNF mRNAs containing novel 5′ exons is regulated by kainic acid 1, 3, 6, 12, and 24 hr after drug administration. The results revealed differential regulation patterns for BDNF transcripts. BDNF exon I and IV transcripts (exons I and III according to Timmusk et al., 1993) have previously been characterized as the most highly induced BDNF mRNAs in response to kainic acid treatment. It was remarkable that in our experiments not only were these BDNF transcripts induced by kainate but also the levels of novel exon V, VII, VIII, and IXA mRNAs were up-regulated peaking at 3–6 hr posttreatment and rapidly down-regulated to basal levels thereafter (Fig. 5). The levels of exon IV transcripts remained elevated at 3–24 hr posttreatment. BDNF transcripts with exon IIA, IIB, and IIC exhibited differential expression profiles in response to kainite treatment. The levels of exon IIC transcripts were markedly elevated at 3 hr, peaked at 6 hr, and decreased at 12–24 hr after kainate treatment. Expression levels of exon 2A and exon 2B transcripts increased moderately at 3 hr, dropped at 6 hr, and reached basal levels at 24 hr posttreatment (Fig. 5). In contrast, the expression levels of BDNF exon III and exon VI mRNAs did not change at any time point studied (Fig. 5). These results agree with the previous reports on the transcript-specific regulation of rat BDNF mRNAs in response to kainate-induced seizures (Timmusk et al., 1993; Sathanoori et al., 2004) and provide the first evidence that the novel BDNF mRNAs are differentially regulated by kainic acid. Our data strongly suggest that as yet unexplored regulatory elements within BDNF gene contribute to the activity-dependent regulation of BDNF mRNA expression.

### Antisense-BDNF Transcripts Are Not Expressed in Mouse and Rat

It was shown recently (Liu et al., 2005) that protein noncoding antisense transcripts are expressed from human BDNF gene locus. Analyses of mouse and rat BDNF gene loci with the AntiHunter software tool (Lavorgna et al., 2004) did not reveal any BDNF antisense transcripts from EST databases. Moreover, alignment of human antisense BDNF exons sequences with mouse and rat EST databases at NCBI did not reveal any rodent ESTs homologous to human antisense BDNF transcripts. Failure to find antisense ESTs transcribed from mouse and rat BDNF gene loci could be explained by the fact that, although EST databases are growing rapidly, they are still undersampling the full mammalian transcriptome. Therefore, we aligned the sequences of human antisense BDNF exons with the respective regions of mouse and rat BDNF genomic sequence. Interestingly, sequences with significant homology to human antisense exons, though present in chimpanzee genome, were missing from mouse and rat genomes. RT-PCR analysis with mouse- and rat-specific primers annealing to the very short regions of homology with human antisense transcripts failed to detect expression of antisense BDNF transcripts in mouse and rat tissues. Therefore, we concluded that antisense BDNF transcripts are human- or primate-specific, as was proposed earlier by Liu and colleagues (2005).

## DISCUSSION

Since the purification of BDNF protein, definitive evidence has emerged for its central role in mammalian brain development, physiology, and pathology. However, the structural organization of rodent BDNF gene has not been revisited since four 5′ exons were first discovered and nomenclature of exons established for rat BDNF gene (Timmusk et al., 1993). This numeration of BDNF exons is currently widely used by the scientific community. In the present work, we show, however, that mouse and rat BDNF gene structure is much more complex than was accepted before. According to our data, mouse and rat BDNF genes consist of a common 3′ exon that encodes the pro-BDNF protein and at least eight 5′ noncoding exons (exons I–VIII). In each BDNF transcript, one 5′ exon is spliced to the protein coding exon. All 5′ exons are controlled by distinct promoters as evidenced by our RACE analysis of the 5′ ends of these exons, as well as expression analysis data. In addition, we identified a novel BDNF transcript both in mouse and in rat that contains only exon IXA, the 5′ extended protein coding exon. Here we suggest a new numbering system for mouse and rat BDNF exons. With regard to the old nomenclature (Timmusk et al., 1993), former exon III corresponds to exon IV, previous exon IV is now exon VI, and the coding exon previously called exon V is now exon IX.

Pro-BDNF, a 32-kDa precursor, undergoes cleavage to release mature 14-kDa BDNF protein as well as a minor truncated form of the precursor (28 kDa). Secreted pro-BDNF activates a heteromeric receptor complex of p75 and sortilin to initiate cell death (Teng et al., 2005) and binds to p75 in hippocampal neurons to enhance long-term depression (Woo et al., 2005). Studies suggest that proneurotrophins account for a significant amount of the total neurotrophins secreted extracellularly, particularly in CNS neurons (Farhadi et al., 2000; Mowla et al., 2001). In mouse, rat, and human, exon I transcripts contain an in-frame AUG that can serve as an alternative translation initiation codon, extending the prepro- region of BDNF by eight amino acids (Timmusk et al., 1993). It can be hypothesized that additional amino acids in the N-terminus of prepro-BDNF can affect the intracellular trafficking of BDNF and play a role in pro-BDNF secretion. In human, BDNF 5′ exons VIB and VII (according to Liu et al., 2005) can contribute to alternative BDNF protein isoforms, because exon VIB can add 15 amino acids to the N-terminus of prepro-BDNF, and exon VII can undergo alternative in-frame splicing leading to the mature BDNF protein isoform that lacks 48 amino acids internally (Liu et al., 2005). None of the novel rodent BDNF exons includes an in-frame ATG, predicting that for these transcripts translation is initiated from the BDNF coding exon.

BDNF is the most abundant and widely distributed neurotrophin in the mammalian CNS. In addition to refining expression patterns of BDNF transcripts that have been identified earlier, results of this study also show that mouse and rat BDNF novel exons III, V, VII, VIII, and IXA are differentially expressed in adult brain and in peripheral tissues. In general, exons that are closely located in the genome are expressed in a similar manner: exons I, II, and III have brain-enriched expression patterns and exons IV, V, and VI are widely expressed also in nonneural tissues. However, 5′ RACE analysis of transcription initiation sites of rat and mouse BDNF new exons and in silico analysis of the regions upstream of these exons (data not shown) suggest that their expression is driven by distinct novel tissue-specific and development- and activity-regulated promoters.

It has been established earlier by using different cellular and animal models that BDNF gene is regulated by neural activity through calcium-mediated pathways (Shieh and Ghosh, 1999; West et al., 2001; Mellstrom et al., 2004) and that BDNF transcripts containing exons I, II, and IV are differentially regulated. BDNF exon I and exon IV transcripts (exons I and III according to Timmusk et al., 1993) have previously been characterized as the most highly induced BDNF mRNAs in response to kainate treatment and KCl-mediated membrane depolarization in embryonic cortical neuron cultures (Tao et al., 1998). Several calcium-responsive elements and transcription factors binding to these elements have been characterized in the promoter regions upstream of these exons (Timmusk et al., 1999; Tabuchi et al., 2002; Tao et al., 2002; Chen et al., 2003b). Here we show that BDNF exon V, exon VII, exon VIII, and exon IXA transcripts are also regulated by kainic acid and that the induction magnitude is comparable to that of BDNF exon I and IV transcripts. In light of our findings, it is attractive to speculate that differential regulation of nine BDNF exon mRNAs would become apparent in different neurodegenerative diseases in which BDNF levels are altered (Phillips et al., 1991; Mogi et al., 1999; Parain et al., 1999; Zuccato et al., 2001). Also, differential regulation of BDNF mRNAs can take place for example in depression, stress, exercise, and learning (Cotman and Berchtold, 2002; Tyler et al., 2002; Hashimoto et al., 2004; Russo-Neustadt and Chen, 2005). Future characterization of the regulatory sequences and transcription factors mediating regulation of novel BDNF transcripts in different disease models is important for understanding BDNF gene regulation and its contribution to pathology.

The role of chromatin remodeling in the activity of different BDNF promoters has been investigated in several recent studies. Neuronal activity-dependent activation of BDNF gene is mediated by decreased CpG methylation of *BDNF* promoter IV and release of a repressor complex containing methyl-cytosine binding protein MeCP2, histone deacetylases HDAC1 and HDAC2, and corepressor mSin3A (Chen et al., 2003a; Martinowich et al., 2003). It has also been shown that histone modifications at specific BDNF promoters are involved in chromatin remodeling during electroconvulsive seizures (Tsankova et al., 2004) and cocaine-induced plasticity (Kumar et al., 2005) in rat and in a mouse model of depression and antidepressant treatment (Tsankova et al., 2006). In addition, zinc finger transcription factor REST/NRSF (Chong et al., 1995; Schoenherr and Anderson, 1995), which recruits multiple cofactors including HDAC1, HDAC2, and mSin3A (for review see Ballas and Mandel, 2005) to repress its target genes, negatively regulates BDNF gene expression by binding to NRSE/RE1 element in BDNF promoter II (Palm et al., 1998; Timmusk et al., 1999; Bruce et al., 2004; Ballas et al., 2005). The present study showed that the DNA demethylating agent 5AzadC evoked robust activation of BDNF gene expression in C6 rat glioma cells and more moderate activation in Neuro2A mouse neuroblastoma cells in a transcript-specific manner: induction of exon I, III, IV, V, VIII, and IXA mRNAs was observed in C6 cells, whereas only exon I and exon III mRNA levels increased in Neuro2A cells. Furthermore, in C6 cells, inhibition of histone deacetylation by TSA up-regulated the levels of BDNF exon III, exon VII, and exon IX transcripts. The results presented in this study suggest the contribution of histone modifications and methylation of BDNF promoters to the regulation of BDNF gene transcription and open up possibilities for addressing these phenomena in more detail.

Finally, we report that, in contrast with the human BDNF gene locus (Liu et al., 2005), mouse and rat BDNF gene loci do not encode antisense mRNA transcripts. These findings demonstrate that regulation of BDNF gene expression by antisense-BDNF transcripts clearly is a human- or primate-specific phenomenon and suggest that regulation of rodent and human BDNF gene differs substantially. Human-specific antisense transcripts have been reported for the tumor suppressor gene ret finger protein 2 (RFP2; Baranova et al., 2003) and for the human protocadherin (PCDH) locus (Lipovich et al., 2006). BDNF has important roles in development, particularly of the nervous system, and plays a central role in brain plasticity-related processes, underscoring the possible role of antisense BDNF gene in regulation of BDNF expression across primates manifesting in specific behavioral phenotypes.

During the preparation of this paper, an article by Liu and colleagues examining the gene structure and expression of BDNF in rodents was published (Liu et al., 2006). However, our study increases the understanding of rodent BDNF gene loci, in that we present several novel data that are complementary to the results of Liu and colleagues. 1) We identified an additional 5′ exon, exon V that was not been reported by Liu et al. Thus, both mouse and rat BDNF genes consist of at least eight 5′ exons spliced to the 3′ coding exon. In addition, we identified a novel BDNF transcript, exon IXA mRNA, consisting of only the 5′ extended protein coding exon. 2) We determined the transcription initiation sites for novel exons (III, V, VII, VIII, and IXA), showing that these exons are transcribed from distinct promoters. 3) Our data show that exon VIII (exon VII according to Liu et al.) is driven by a separate promoter. Liu and colleagues' data argue that transcripts containing exons VII and VIII (exons VI and VII according to Liu et al.) share the same promoter. 4) Our expression analysis data for all BDNF transcripts includes a wider range of tissues and brain structures analyzed both in rat and in mouse. 5) Liu et al. studied the regulation of some BDNF transcript expression in brain upon administration of cocaine. Our data show activity-dependent regulation of rat BDNF mRNAs by kainic acid-induced seizures in rat hippocampus. Moreover, we report differential regulation of the expression of BDNF transcripts by DNA methylation and histone deacetylation. Taken together, the results of the present study on mouse and rat BDNF gene structure and tissue-specific expression provide new challenges and opportunities to identify mechanisms regulating the activity of novel BDNF promoters that contribute to the expression levels of BDNF and possibly also to the changes in BDNF expression in neurodegenerative and neuropsychiatric disorders.
